# Assessment of Antimicrobial Transition Errors from Hospitals to Skilled Nursing Facilities

**DOI:** 10.1093/ofid/ofag111

**Published:** 2026-03-10

**Authors:** Amy Y Kang, Guarina A Garcia Delgado, Ashley Nguyen, Maddie Yeh, Tracy Ly, Richard Beuttler, Abisay Ortega, Donna Phan Tran, Evelyn Flores, Charis Tjoeng, Andrew Bishop, Praneet Kalkat, Loren G Miller

**Affiliations:** Department of Pharmacy Practice, Chapman University School of Pharmacy, Irvine, California, USA; Department of Pharmacy, Harbor-UCLA Medical Center, Torrance, California, USA; Division of Infectious Diseases, Lundquist Institute at Harbor-UCLA Medical Center, Torrance, California, USA; Division of Infectious Diseases, Lundquist Institute at Harbor-UCLA Medical Center, Torrance, California, USA; Division of Infectious Diseases, Lundquist Institute at Harbor-UCLA Medical Center, Torrance, California, USA; Division of Infectious Diseases, Lundquist Institute at Harbor-UCLA Medical Center, Torrance, California, USA; Department of Pharmacy, Kaiser Permanente, Fontana, California, USA; Department of Pharmacy Practice, Chapman University School of Pharmacy, Irvine, California, USA; Division of Infectious Diseases, Lundquist Institute at Harbor-UCLA Medical Center, Torrance, California, USA; Division of Infectious Diseases, Lundquist Institute at Harbor-UCLA Medical Center, Torrance, California, USA; Division of Infectious Diseases, Lundquist Institute at Harbor-UCLA Medical Center, Torrance, California, USA; Division of Infectious Diseases, Department of Medicine, Harbor-UCLA Medical Center, Torrance, California, USA; Division of Infectious Diseases, Department of Medicine, Providence Regional Medical Center Everett, Everett, Washington, USA; Division of Infectious Diseases, Department of Medicine, Harbor-UCLA Medical Center, Torrance, California, USA; Division of Infectious Diseases, Lundquist Institute at Harbor-UCLA Medical Center, Torrance, California, USA; Division of Infectious Diseases, Department of Medicine, Harbor-UCLA Medical Center, Torrance, California, USA; Department of Medicine, David Geffen School of Medicine at UCLA, Los Angeles, California, USA

## Abstract

**Background:**

More than 1 million Americans reside in skilled nursing facilities (SNFs). Antimicrobial transition errors among patients transferred from hospital to SNFs pose safety risks and may lead to poor outcomes, but data on such errors are limited.

**Methods:**

We conducted a retrospective cohort study of infectious diseases clinics from 1 June 2020 through 30 November 2023 at the Los Angeles County Department of Health Services, a large safety-net health system. We performed logistic regression analyses to identify factors associated with antimicrobial transition errors and poor infection outcomes.

**Results:**

We screened records of 6865 clinic patients, among whom 112 were SNF residents who were receiving post–hospital discharge antimicrobials. Mean age was 62 years, 37% were female, and 57% were Hispanic/Latino. Transition errors occurred in 32 (29%) patients. Common medications associated with errors were penicillin class (39%), tetracycline class (38%), and daptomycin (36%). In our multivariable model, age, Charlson Comorbidity Index score, number of medications, Centers for Medicare & Medicaid Services SNF rating, and therapy duration were not significantly associated with transition errors. Older age was the only independent predictor of poor infection outcome (*P* & .02). There was a nonsignificant trend between antimicrobial transition errors and poor infection outcome (odds ratio, 1.63 [95% confidence interval, .58–4.81]).

**Conclusions:**

Nearly one-third of patients transitioning from hospitals to SNFs on antimicrobials experienced ≥1 antimicrobial transition error. We did not identify risk factors for antimicrobial transition errors. The trend toward an association between antibiotic transition errors and poor infection outcomes warrants further investigation in more robust data sets.

## Teaser

In a multicenter survey of infectious diseases clinics, nearly one-third of skilled nursing home residents experienced medication errors posthospitalization. A potential association between antibiotic transition errors and adverse infection outcomes may exist and should be explored further in studies with larger, more robust data sets.

Nearly a quarter of patients who are transitioned from hospitals to skilled nursing facilities (SNFs) are discharged on antimicrobials [1]. Given that >5 million patients are discharged to SNFs annually [2], approximately 1 million hospitalized patients will continue on antimicrobials at time of SNF transfer annually in the United States. Inappropriate changes in medications during SNF care transitions are estimated to be >20% [3]. Antimicrobial transition errors are particularly problematic as antimicrobial treatment is critical to eradicate infections and to prevent infection relapse.

There are limited data on antimicrobial transition errors after SNF transfer. One study by Dickinson and colleagues reported the proportion of transfers associated with transition errors (30% [12/40]) but did not assess factors associated with inappropriate antimicrobial changes during the care transitions or the impact of these errors on clinical outcomes. Additionally, patients in this investigation were all clinical trial participants; thus, findings may not reflect real-world clinical practice [4].

Despite the frequent occurrence of care transitions from hospitals to SNFs and the potential for antimicrobial transition errors, there remains a significant gap in knowledge regarding the factors contributing to these errors and their clinical consequences. This knowledge gap is particularly concerning given that patients requiring complex, continuous management are often discharged to SNFs, a disposition that has been strongly associated with a high 30-day rehospitalization rate [5, 6]. This high rehospitalization rate further emphasizes the medical vulnerability of this population.

Given these challenges, it is crucial to improve our understanding of antimicrobial transition errors to enhance patient safety. To this end, our study sought to address this gap by describing the frequency and types of antimicrobial transition errors, identifying risk factors associated with these errors, and assessing their impact on clinical outcomes.

## METHODS

### Study Design, Population, and Setting

We performed a retrospective cohort study, searching databases within the Los Angeles County Department of Health Services (LAC DHS) system. LAC DHS is the second largest municipal safety-net health system in the United States and is a closed system with a shared electronic health record (EHR). LAC DHS has 3 infectious diseases (ID) clinics at each of the following medical centers: Los Angeles General Medical Center, Harbor-UCLA (University of California, Los Angeles) Medical Center, and Olive View-UCLA Medical Center. We included patients in our study if they met all the following criteria: (1) discharge from the hospital to an SNF between 1 February 2020 and 30 November 2023; (2) had at least 1 follow-up visit with the ID clinic postdischarge; and (3) were prescribed antimicrobial therapy. We excluded patients who did not have care transitions from the hospital to an SNF and patients who left their SNF against medical advice during treatment. If patients had >1 care transition from the hospital to the SNF during the study period, we included only the first transition.

We exclusively screened patients receiving follow-up care at ID clinics to ensure a more accurate identification of antimicrobial transition errors. Due to the specialized focus of ID specialists on antimicrobial treatment and their meticulous review of SNF antimicrobial records, antimicrobial transition errors may be more likely to be detected and documented by ID clinic patients compared to primary care providers who have broader focus encompassing all clinical aspects. Therefore, we strategically screened ID clinic patients exclusively to maximize the chances of catching discrepancies that occurred during the care transitions and minimize the potential of misinterpretation of these discrepancies. For the purpose of this study, we included only general ID clinics and did not include subspecialty ID clinics serving immunocompromised patients such as those with human immunodeficiency virus or hepatitis C infection.

Utilizing a standardized chart abstraction instrument, each medical record was screened and abstracted in duplicate by 2 trained research assistants to ensure accuracy and consistency. Any discrepancies were resolved by discussion. If consensus could not be achieved, a principal investigator (A. Y. K.) served as arbitrator. Approval of the study protocol was obtained from the Institutional Review Board (IRB) at the Lundquist Institute at Harbor-UCLA Medical Center (IRB No. 18CR-32563-01).

### Outcomes Definition

Antimicrobial transition errors were defined as any discrepancy in the antimicrobial therapy regimen(s) that occurred after transferring a patient from a hospital setting to an SNF, which could not be attributed to changes in the patient's clinical status (eg, adjustments for changing renal function). These errors included antimicrobial choice, frequency, route, duration, and monitoring. Antimicrobial choice errors were defined as changes in antimicrobials without clear documentation stating the intention to change, and the changes are not aligned with the prescribed treatment plan as documented in the ID or any of the treating physician's notes. Antimicrobial frequency errors were defined as changes in the frequency of antimicrobial administration without a clinical reason (eg, kidney dysfunction or improvement), including both more frequent and less frequent dosing than prescribed. Antimicrobial route errors were defined as discrepancies in the route of administration, such as planned oral or feeding tube administration, but intravenous (IV) route was administered instead, without a clinical reason. Antimicrobial duration errors were defined as deviations from the planned duration of antimicrobial therapy, including either shorter or longer durations than initially prescribed without documented intentions or clinical reasons, and the changes are not aligned with the patient's clinical treatment plan as documented in the ID or any of the treating physician's notes. For example, if the ID team recommended an antibiotic for 7 days but the discharge prescription was written for 3 days (“shorter than planned”) or 14 days (“longer than planned”), this was classified as a duration transition error. Durations of therapy were assessed using the cumulative days of appropriate inpatient and outpatient therapy and were recorded based on documentation in the patient records, which varied between a specified end date (eg, “end of therapy = 5 November 2025”) and total days of therapy (eg, “14 days”). Accordingly, a margin of ±1 day was applied to duration assessments to allow for minor variability in documentation practices, such as differing conventions on whether the first treatment day was counted as day 0 or day 1. Additional inpatient days of therapy beyond ID sign-off were incorporated into the calculated discharge prescriptions. Monitoring errors were defined as missed monitoring parameters that were recommended for ongoing antimicrobial therapy for safe and effective use, such as monitoring renal function, liver enzymes, or therapeutic drug levels (eg, vancomycin troughs or aminoglycoside levels). These errors were identified through a comprehensive review of medical records, including inpatient physician and provider notes, discharge summaries, ID outpatient clinic notes, and any other pertinent outpatient documents.

Clinical outcomes were separated into 4 categories: cure, failure, indeterminate, and not applicable. Treatment cure was defined as the complete resolution or significant improvement of infection signs and symptoms, such that no further antibacterial therapy was necessary. Treatment failure was defined as unplanned extension of antimicrobials due to lack of infection resolution or infection relapse, unplanned surgical intervention due to lack of infection resolution or infection relapse, infection-related death, or change of therapy due to adverse events. Patients for whom the assessment pertaining to ID was not documented such that patient's clinical response could not be determined were classified as indeterminate. Patients who were receiving antimicrobial suppression therapy indefinitely were classified as not applicable.

### Statistical Analysis

To determine factors associated with antimicrobial transition errors and assess whether poor infection-associated outcomes were linked to antimicrobial transition errors, we performed a priori power calculations to ensure robust logistic multivariate models with ≥10 outcomes per predictor variable [7]. Based on literature estimating an antimicrobial transition error rate of 30% [4] and chart review data from a single LAC DHS site indicating an average of 60 patients discharged to SNFs yearly with follow-up in ID clinics, we estimated that data extraction from a 3-year period with 4 hospital sites would provide adequate power for our multivariable models (a total of 4 sites × 60 patients per year × 3 years = 720 patients). We then anticipated (720 × 30%) = 216 antimicrobial transition errors. For the model of treatment failure, with an estimated 25% treatment failure rate based on literature that shows 24% of patients who are discharged on IV antimicrobial experience complications during treatment course [8, 9] and 18.5% require unplanned hospitalization [10], we anticipated 180 treatment failures.

The frequency and types of the antimicrobial transition errors were analyzed descriptively. For a comparative analysis of patients with versus without antimicrobial transition errors, we utilized χ^2^ test or Fisher exact test for categorical variables and Student *t* test or Mann–Whitney test for continuous variables, as appropriate. To identify risk factors associated with antimicrobial transition errors, we developed a multivariate logistic regression model utilizing preselected independent predictor variables including patient age, Charlson Comorbidity Index (CCI) score, the number of scheduled medications, planned days of therapy at SNF postdischarge, and the Centers for Medicare & Medicaid Services’ (CMS) Five-Star Nursing Home Quality Rating System. Additionally, in a similar manner, we developed a separate multivariate logistic regression model to identify factors associated with treatment failure. Patients whose clinical outcomes were classified as indeterminate or not applicable were excluded from this analysis. The preselected independent predictor variables included antimicrobial transition errors, age, CCI score, and CMS's Five-Star Nursing Home Quality Rating System. A *P* value of ≤.05 was regarded as significant. All data analysis was performed using Stata version 18.0, GraphPad Prism version 10.2.3, and R version 4.4.0 software.

## RESULTS

We screened 6865 patients and identified 112 patients who met our study criteria. All patients included in our study were seen in consultation by the inpatient ID service during their hospitalization and subsequently had follow-up scheduled in the ID clinic. All patients excluded from our analysis were excluded because they were discharged to settings other than SNFs or represented duplicate patient records. Of these 112 patients, 32 (29%) experienced at least 1 transition error. The demographic characteristics of the study population are summarized in Table 1. Overall, patients’ mean age was 62 years, and the majority of patients were male (63%) and Hispanic/Latino (57%). The most common comorbidities were diabetes (51%) and the majority of infection diagnoses were bone and joint infection (63%).

**Table 1.** Demographics of Patients Seen in Infectious Diseases Clinics on Antibiotics Who Were Transitioned From Hospital to a Skilled Nursing Facility

Characteristic	Total (n = 112)	Antimicrobial Transition Errors (n = 32)	No Antimicrobial Transition Errors (n = 80)	*P* Value
Age, y, mean (SD)	62 (12)	61 (11)	63 (13)	.61
Sex				.52
Male	71 (63)	22 (69)	49 (61)	
Female	41 (37)	10 (31)	31 (39)	
Race				.40
African American/Black	15 (13)	5 (16)	10 (13)	
American Indian/Alaska Native	1 (1)	0 (0)	1 (1)	
Asian	9 (8)	4 (13)	5 (6)	
Hispanic/Latino	64 (57)	16 (50)	48 (60)	
White	15 (13)	5 (16)	10 (13)	
Multiracial	1 (1)	0 (0)	1 (14)	
Other	4 (4)	1 (3)	3 (4)	
Unsure/unclear	3 (3)	0 (0)	3 (4)	
Preferred English				1.0
English	60 (54)	17 (53)	43 (54)	
Not English	52 (46)	15 (46)	37 (46)	
Immunocompromising condition	8 (7)	2 (6)	6 (8)	1.0
HIV with CD4 count ≤200 cells/μL	1 (1)	0 (0)	1 (1)	
Chemotherapy within prior 6 mo to infection diagnosis	4 (4)	2 (6)	3 (4)	
Immunomodulatory therapy or corticosteroids (prednisone ≥20 mg, equivalent dose) for ≥14 d	2 (2)	1 (3)	2 (3)	
Organ transplant or HSCT	1 (1)	0 (0)	1 (1)	
Substance use disorder	18 (16)	2 (6)	16 (20)	.09
Diabetes^a^	57 (51)	16 (50)	41 (51)	.13
Controlled diabetes	31 (28)	10 (31)	21 (26)	
Uncontrolled diabetes	15 (13)	1 (3)	14 (18)	
No HbA1c value available	11 (10)	5 (16)	6 (8)	
CCI score, median (IQR)	4 (3–6)	4 (3–5)	4 (3–6)	.27
Infection diagnosis^b^				.15
Catheter-associated	4 (4)	3 (9)	1 (1)	
Endocarditis	9 (8)	1 (3)	8 (10)	
Gastrointestinal	12 (11)	4 (13)	8 (10)	
SSTI	19 (17)	7 (22)	12 (15)	
Bone and joint infection	71 (63)	25 (78)	46 (58)	
Pulmonary infection	8 (7)	2 (6)	6 (8)	
Urinary	9 (8)	0 (0)	9 (11)	
CNS	19 (17)	5 (16)	14 (18)	
ICU admission during the hospitalization	26 (23)	6 (19)	20 (25)	.62
Scheduled medications, median (IQR)	12 (8–15)	13 (7–17)	12 (8–15)	.50
As-needed medications, median (IQR)	3 (1–5)	3 (2–5)	2 (1–5)	.26
Days of inpatient therapy, median (IQR)	7 (3–14)	5 (2–5)	7 (4–14)	.51
Days of therapy at SNF postdischarge, median (IQR)	31 (25–40)	30 (20–42)	31 (27–40)	.72

Data are presented as No. (%) unless otherwise indicated.

Abbreviations: CNS, central nervous system; HbA1c, hemoglobin A1c; HIV, human immunodeficiency virus; HSCT, hematopoietic stem cell transplant; ICU, intensive care unit; IQR, interquartile range; SD, standard deviation; SNF, skilled nursing facility; SSTI, skin and soft tissue infection.

^a^Controlled diabetes was defined as HbA1c <8% or documented in clinical notes as meeting individualized glycemic targets. Uncontrolled diabetes was defined as not meeting the criteria for controlled diabetes.

^b^Some patients had >1 infection diagnosis.

Of the 32 patients who experienced antimicrobial transition errors, a total of 36 errors were identified, with some patients having >1 error during their transition. The majority of errors were classified as antimicrobial duration errors (30/36 [83%]), with most being shorter than planned (29/36 [81%]) and 1 being longer than planned (1/36 [3%]). Errors related to antimicrobial frequency were identified in 2 of the 36 errors (6%), both involving longer-than-planned intervals. Similarly, 2 of the 36 errors (6%) were related to route of administration, where the planned route of oral or feeding tube administration was substituted with IV administration. Finally, 2 of the 36 errors (6%) were categorized as inappropriate antimicrobial choice, where the discharge regimen deviated from the final ID recommendation without clinical or microbiologic justification (Table 2).

**Table 2.** Antimicrobial Transition Error Classification^a^

Type of Error (n = 36)	No. (%)
Antimicrobial duration	30 (83)
Shorter duration than planned	29 (81)
Longer duration than planned	1 (3)
Antimicrobial frequency	2 (6)
Longer frequency than planned	2 (6)
Antimicrobial route	2 (6)
Oral/feeding tube route planned, IV route administered	2 (6)
Antimicrobial choice	2 (6)

Abbreviation: IV, intravenous.

^a^A total of 32 patients experienced antimicrobial transition errors, with a total of 36 errors identified, as some patients had >1 error during the transition.

Out of the 153 total medication orders reviewed in all included patients, 36 (24%) were associated with errors (Table 3). Among these errors, the most common antimicrobial class was the penicillin class (12/31 [39%]), with oxacillin-nafcillin and amoxicillin showing error rates of 67% (2/3) and 33% (1/3), respectively. Among carbapenems, over half (9/17 [53%]) were associated with errors, including ertapenem (5/10 [50%]), meropenem (3/6 [50%]), and a single error involving imipenem (1/1 [100%]). Cephalosporins were commonly used (39/153 [26%]) and had a relatively low overall error rate (6/39 [15%]). However, certain agents, such as ceftriaxone, showed higher error rates (5/22 [23%]). Errors associated with daptomycin (5/14 [36%]) and tetracycline-class agents (3/8 [38%]) were also notable, though the 100% (1/1) error rates observed for omadacycline and minocycline (1/1) were based on single incidents for each agent. Infrequent errors were also noted with trimethoprim-sulfamethoxazole (1/6 [17%]), vancomycin (1/12 [8%]), and rifamycin (1/12 [8%]). Antifungal treatments errors occurred in 17% (3/18), with azoles being the main contributors (3/15 [20%]). Administration route was a key factor, with IV medications having a higher error rate compared to the oral route (29% [24/83] vs 17% [12/70]). Dosing frequency showed the highest errors with a single incidence of every 4 hours regimen (1/1 [100%]), followed by every 6 hours (1/2 [50%]) and every 48 hours (2/5 [40%]).

**Table 3.** Medications Associated With Antimicrobial Transition Errors in Comparison to Total Medication Orders^a^

Antimicrobial Regimen	Antimicrobial Transition Errors (n = 36)/ Total Antimicrobial Orders (n = 153), No. (%)
Antibacterials	33/137 (24)
Penicillin class	12/31 (39)
Oxacillin-nafcillin	2/3 (67)
Amoxicillin	1/3 (33)
Carbapenems	9/17 (53)
Ertapenem	5/10 (50)
Imipenem	1/1 (100)
Meropenem	3/6 (50)
Cephalosporins	6/39 (15)
Ceftriaxone	5/22 (23)
Ceftaroline	1/1 (100)
Daptomycin	5/14 (36)
Linezolid	1/3 (33)
Tetracycline class	3/8 (38)
Doxycycline	1/5 (20)
Omadacycline	1/1 (100)
Minocycline	1/1 (100)
Fluoroquinolones	3/14 (21)
Ciprofloxacin	2/10 (20)
Levofloxacin	1/3 (33)
TMP-SMX	1/6 (17)
Vancomycin	1/12 (8)
Other	1/15 (6)
Rifamycin	1/12 (8)
Antifungals	3/18 (17)
Azoles	3/15 (20)
Fluconazole	2/10 (20)
Isavuconazole	1/2 (50)
Route	
IV	24/83 (29)
PO	12/70 (17)
Frequency	
Every 4 h	1/1 (100)
Every 6 h	1/2 (50)
Every 8 h	6/18 (33)
Every 12 h	8/56 (14)
Every 24 h	18/71 (25)
Every 48 h	2/5 (40)

Abbreviations: IV, intravenous; PO, oral; TMP-SMX, trimethoprim-sulfamethoxazole.

^a^A total of 32 patients experienced antimicrobial transition errors, with a total of 36 errors identified, as some patients had >1 error during the transition.

The overall clinical cure rate was 38% (42/112), with a lower cure rate observed in patients with antimicrobial transition errors compared to those without (28% vs 41%, *P* = .28) (Table 4). Lack of infection resolution or relapse led to an unplanned extension of antimicrobials in 33% of patients, with higher rates in the transition error group compared to the no-error group (38% vs 31%). The overall mortality rate was low (2/112 [2%]) and no deaths were reported in the transition error group. The need for unplanned surgical intervention due to lack of infection resolution or relapse was rare, occurring in only 4 (4%) patients. Additionally, adverse events leading to a change in therapy were minimal, reported in only 1 patient from the no antimicrobial transition errors group (1%). Indeterminate outcomes, including patients lost to follow-up or cases where clinical response could not be assessed, accounted for 7% (8/112) overall.

**Table 4.** Clinical Outcomes of Patients With and Without Antimicrobial Transition Errors

Outcome	Total (n = 112)	Antimicrobial Transition Errors (n = 32)	No Antimicrobial Transition Errors (n = 80)	*P* Value
Clinical cure achieved				.28
Yes	42 (38)	9 (28)	33 (41)	
No	44 (39)	15 (47)	29 (36)	
Death	2 (2)	0 (0)	2 (3)	
Unplanned extension of antibiotics due to lack of infection resolution or infection relapse	37 (33)	12 (38)	25 (31)	
Unplanned surgical intervention due to lack of infection resolution or infection relapse	4 (4)	3 (1)	1 (1)	
Change of therapy due to adverse events	1 (1)	0 (0)	1 (1)	
Indeterminate	8 (7)	1 (3)	7 (9)	
Lost to follow-up	6 (5)	1 (3)	5 (6)	
Assessment pertaining to infectious diseases not documented such that patient's clinical response could not be determined	2 (2)	0 (0)	2 (3)	
Not applicable; patient is receiving suppression antibiotic life-long	18 (16)	7 (22)	11 (14)	

Data are presented as No. (%).

In our multivariable regression analysis of antimicrobial transition errors that included age, CCI score, number of scheduled medications, CMS rating of the SNF, and days of therapy at the SNF, no variables were identified to be an independent predictor (Table 5).

**Table 5.** Multivariable Regression Analysis of Factors Associated With Antimicrobial Transition Errors and Clinical Failure

Variable	Odds Ratio	(95% CI)	*P* Value
Antimicrobial transition errors			
Age	0.98	(.92–1.04)	.47
CCI score	0.86	(.60–1.17)	.35
Number of scheduled medications	1.01	(.90–1.13)	.90
CMS rating of SNF	0.78	(.40–1.44)	.44
Days of therapy at SNF	0.99	(.96–1.02)	.70
Clinical failure			
Antimicrobial transition errors	1.63	(.58–4.82)	.36
Age	1.06	(1.01–1.13)	.02
CCI score	0.97	(.78–1.22)	.80
CMS rating of SNF	0.94	(.57–1.56)	.81

Abbreviations: CCI, Charlson Comorbidity Index; CI, confidence interval; CMS, Centers for Medicare & Medicaid Services; SNF, skilled nursing facility.

In our multivariable regression model of clinical failure, we found that age was significantly associated with poor outcomes (odds ratio [OR], 1.06 [95% confidence interval {CI}, 1.01–1.13]; *P* = .02), but there was no statistically significant association with antimicrobial transition errors (OR, 1.63 [95% CI, .58–4.82]; *P* = .36) or other variables in the model (Table 5).

## DISCUSSION

We found that nearly 1 in 3 patients in our healthcare system who were seen in ID clinics after being discharged to an SNF experienced an antimicrobial transition error. These findings highlight the complexity and challenges associated with managing antimicrobial regimens during care transitions. The most prevalent type of error in our study was related to the duration of antimicrobial therapy, the vast majority of which were shorter than planned therapy duration. This high rate raises concerns about the potential for incomplete treatment course and potential adverse outcomes. Conversely, longer-than-planned durations, although much less common, pose risks for adverse drug events and unnecessary healthcare costs. Errors related to antimicrobial frequency and route were also notable. Errors involving the administration frequency and inappropriate changes in the route of administration highlight the need for clear communication and documentation during transitions.

General patient care medication transition errors involving a medication discrepancy have been previously studied across various healthcare settings, particularly during transitions from hospital to SNFs [3, 11–17]. In acute care discharge scenarios, studies have shown that medication reconciliation errors can occur in up to 71%–78% of patients transitioning to post–acute care facilities [14, 16], often driven by inadequate communication between healthcare teams or incomplete discharge instructions [3, 11–13, 16].

Antimicrobial transition errors are especially critical due to their potential to cause immediate and far-reaching consequences that extend beyond the individual patient. Errors, such as shortened or prolonged durations, incorrect administration routes, or inappropriate antimicrobial choices, lead to severe adverse drug event and higher healthcare expenditures, and potentially incomplete pathogen eradication, eventually leading to treatment failure, or the development of resistant organisms [18–20]. These untoward outcomes not only jeopardize patient recovery but also pose public health risks by potentially enabling the spread of resistant pathogens. Importantly, antimicrobial therapy is most often prescribed for acute infections, where timely and precise management is critical, making transition errors particularly impactful. Taken together, the acute nature of infections, the complexity of antimicrobial regimens, and the broader public health implications underscore why antimicrobial transition errors warrant heightened attention and dedicated efforts to improve care transitions.

Our study found a higher rate of errors in IV therapy orders (30%) compared to oral therapies (17%), likely due to the greater complexity of IV regimens. IV therapies require careful dosing, infusion management, and monitoring, increasing risks of miscommunication and documentation errors during transitions [21]. To reduce these errors, standardized transitions of care protocols for IV therapy, improved multidisciplinary collaboration, and EHR decision support tailored to IV medications are needed to enhance patient safety and care continuity.

At our health system, the hospital-to-SNF transition and discharge prescribing processes are largely managed by the primary medical teams. Although pharmacists and nurses may be involved in discharge planning, their participation in antimicrobial-specific reconciliation and discharge prescription review is not standardized. Structured interventions, particularly pharmacist-led medication reconciliation programs, have shown significant potential to reduce medication transition errors and improve patient outcomes. Gillespie et al demonstrated an 80% reduction in medication-related readmissions in patients aged ≥80 years who received care from ward-based pharmacists [22]. Similarly, a quasi-experimental study found that medication reconciliation for patients transitioning from SNFs resulted in a 78% reduction in mortality within 60 days postdischarge [23]. A retrospective study also highlighted the role of pharmacists, who identified 33 medication errors in 324 discharged patients; 76% of pharmacist-recommended interventions were accepted by healthcare providers, showcasing their impact in supporting safe transitions [17]. A pragmatic, prospective controlled trial in Slovenia found that routine pharmacist-led medication reconciliation reduced clinically important discharge errors from 62% to 9% [24]. Ongoing efforts, such as the Pharmacy Integrated Transitions (PIT) trial, continue to evaluate the effectiveness of pharmacist involvement in improving care transitions [25]. Collectively, these findings emphasize the critical role of pharmacists and medication reconciliation in enhancing safety during transitional care. Although it is not well-established which classes of medications pose the greatest patient safety risk when associated with antimicrobial transition errors, our data suggest that antimicrobial transition errors have a high risk for potential to cause harm.

In addition to the broader medication reconciliation literature, several studies have examined antibiotic prescribing at hospital discharge, including the subset of patients discharged to SNFs. Parsels et al identified hospital discharge as a key “missed opportunity” for antimicrobial stewardship, noting that an ID pharmacist's review of discharge oral antimicrobial prescriptions sent to our hospital-operated outpatient pharmacy resulted in identification of a substantial number of drug-related problems leading to subsequent interventions [26]. One systemic review demonstrated that focused antibiotic stewardship interventions at discharge—particularly electronic decision-support tools, discharge-specific algorithms, targeted review or discharge advice in discharge prescriptions by ID pharmacists, and education to healthcare professionals—can improve discharge antibiotic prescribing [27]. Together, these data support our finding that antimicrobial transition errors are common and highlight ID pharmacist–driven stewardship as an important solution to improve the discharge process, including discharges to SNFs.

Our analysis did not identify any significant predictors of antimicrobial transition errors in our model that included age, CCI score, number of scheduled medications, CMS SNF rating, and days of therapy at the SNF. This lack of significant associations suggests that no single factor stands out as a priority when addressing transition errors. Instead, it underscores the need for careful management of all patients to ensure error-free transitions from hospitals to SNFs.

Formulary variation between hospitals and receiving SNFs, as well as insurance-driven restrictions, may also play a role in antimicrobial choice errors. We did not closely examine formulary status. However, 2 antibiotics involved in antibiotic choice errors were daptomycin and ertapenem, which are sometimes limited by formulary or coverage constraints in SNFs, suggesting that back-and-forth adjustments between teams could have been a contributing mechanism. Additionally, clinical outcomes in general are inherently multifactorial and cannot be ascribed to any single factor; antimicrobial transition errors, underlying comorbidities, severity of illness, adequacy of source control, and other host-related factors could have acted in combination to influence poor outcomes. More robust investigations may be helpful at identifying those factors most influential at driving poor outcomes. In our multivariable analysis, older age was the only independent predictor of poor infection outcome, likely reflecting underlying frailty and comorbidity.

Although our study found that patients with antimicrobial transition errors had numerically higher rates of poor clinical outcomes compared to those without errors, these differences were not statistically significant. Specifically, patients with transition errors had lower rates of clinical cure at the end of the planned therapy (25% vs 40%) and higher rates of unplanned extensions of antimicrobial, unplanned surgical interventions, and adverse reactions. The lack of statistical significance may be due to the smaller than anticipated sample size. Our a priori power calculations overestimated the number of patients seen in ID clinics on antibiotics (720 anticipated, but only 112 seen) and thus overestimated the number of transition errors (216 anticipated but only 32 seen). Recognizing this limitation, we conducted post hoc power calculations to determine the sample size required to detect a significant association of the magnitude we observed with 80% power, should such an effect exist. Our calculation indicated that a total sample size of 579 patients would be necessary to achieve the desired power. Thus, our current study was likely underpowered to detect smaller, yet clinically meaningful, effects of the antimicrobial transition errors on poor infection outcomes. However, our screening methodology, which focused on ID clinic patients, very likely excluded a broader population of patients discharged to SNFs on antibiotics and managed by primary care providers or other specialties. Our approach, though allowing for detailed analysis within a specialized patient subset, may have unintentionally underestimated the prevalence and impact of antimicrobial transition errors in general.

The findings from this study have several important clinical implications. First, healthcare providers should be aware of the high risk of antimicrobial transition errors and prioritize interventions to improve the accuracy of antimicrobial regimens during care transitions. This includes ensuring thorough medication reconciliation at discharge, providing clear and detailed discharge instructions, and fostering effective communication between hospital and SNF staff. Second, our results highlight the need for ongoing education and training for healthcare providers on best practices for antimicrobial management during care transitions. This training should emphasize the importance of accurate documentation, appropriate duration and frequency of antimicrobial therapy, and the correct route of administration. Finally, the development and implementation of standardized protocols for antimicrobial transitions could help reduce the occurrence of errors. These protocols should include checklists for discharge planning, EHR tools to flag potential errors, and postdischarge follow-up to monitor and address any issues that arise.

This study has several limitations. First, the retrospective nature of the study has its inherent bias and limitations. Second, our decision to screen only ID clinic patients likely contributed to the small sample size and may have introduced selection bias. While ID specialists are more likely to detect and document antimicrobial transition errors, the exclusion of patients with antimicrobial transitions managed by primary care providers or other specialties may mean that our findings significantly underestimate the true prevalence and spectrum of antimicrobial transition errors in the broader patient population. Third, while our study identified errors in antimicrobial transitions, it did not investigate the underlying causes, such as provider decision-making, communication failures, or system-level issues. This is partly due to the retrospective nature of our study, which makes it challenging to uncover the contextual factors behind these errors. Future prospective studies are better suited to explore these root causes and provide insights for targeted interventions. Fourth, there may be unmeasured confounding factors that influenced the occurrence of antimicrobial transition errors and patient outcomes. For example, variations in hospital discharge processes, differences in the experience and training of healthcare providers, and the availability of resources at SNFs could all contribute to the likelihood of errors but were not captured in our analysis.

In summary, our study highlights the prevalence and types of antimicrobial transition errors from hospitals to SNFs and underscores the need for targeted interventions to minimize these errors. While our findings provide valuable insights, further research, particularly with larger and more diverse populations, is needed to fully understand the factors contributing to these errors and to develop effective strategies for preventing them.
